# SMN-deficiency disrupts SERCA2 expression and intracellular Ca^2+^ signaling in cardiomyocytes from SMA mice and patient-derived iPSCs

**DOI:** 10.1186/s13395-020-00232-7

**Published:** 2020-05-08

**Authors:** Guzal Khayrullina, Kasey E. Moritz, James F. Schooley, Naheed Fatima, Coralie Viollet, Nikki M. McCormack, Jeremy T. Smyth, Martin L. Doughty, Clifton L. Dalgard, Thomas P. Flagg, Barrington G. Burnett

**Affiliations:** 1 Department of Anatomy, Physiology and Genetics, F. Edward Hebert School of Medicine, Bethesda, MD USA; 2 The American Genome Center, Uniformed Services University of the Health Sciences, Bethesda, MD USA

## Abstract

Spinal muscular atrophy (SMA) is a neurodegenerative disease characterized by loss of alpha motor neurons and skeletal muscle atrophy. The disease is caused by mutations of the *SMN1* gene that result in reduced functional expression of survival motor neuron (SMN) protein. SMN is ubiquitously expressed, and there have been reports of cardiovascular dysfunction in the most severe SMA patients and animal models of the disease. In this study, we directly assessed the function of cardiomyocytes isolated from a severe SMA model mouse and cardiomyocytes generated from patient-derived IPSCs. Consistent with impaired cardiovascular function at the very early disease stages in mice, heart failure markers such as brain natriuretic peptide were significantly elevated. Functionally, cardiomyocyte relaxation kinetics were markedly slowed and the *T*_50_ for Ca^2+^ sequestration increased to 146 ± 4 ms in SMN-deficient cardiomyocytes from 126 ± 4 ms in wild type cells. Reducing SMN levels in cardiomyocytes from control patient IPSCs slowed calcium reuptake similar to SMA patent-derived cardiac cells. Importantly, restoring SMN increased calcium reuptake rate. Taken together, these results indicate that SMN deficiency impairs cardiomyocyte function at least partially through intracellular Ca^2+^ cycling dysregulation.

## Background

Spinal muscle atrophy (SMA) is one of the leading genetic causes of infant mortality. SMA is caused by survival motor neuron (SMN) protein deficiency due to deletions or mutations of the *SMN1* gene. Individuals also carry two or more copies of a paralog gene, *SMN2*, which produces suboptimal levels of the SMN protein. The amount of full-length protein produced from *SMN2* is an important determinant of disease severity [1, 2]. SMA patients with more than two copies of *SMN2*, as a result of duplication, tend to have less severe forms of the disease. Recently, therapeutic breakthroughs, gene-replacement therapy using viral delivery [3] and altering *SMN2* splicing using antisense oligonucleotides [4], have remarkably improved motor function and survival of SMA patients, but patients remain prone to cardiopulmonary complications leading to opportunistic upper respiratory tract infections [5].

Motor neuron loss and consequent muscle atrophy are the defining features of the SMA phenotype. SMN is ubiquitously expressed in all tissues, where it is known to play a key role in snRNP biogenesis and spliceosome assembly [6–8]. It is unclear why reduced levels of the ubiquitously expressed SMN protein selectively target anterior horn cells of the spinal cord. Since spliceosome assembly occur in all cells, it is possible that reduced SMN function in other tissues may contribute to the clinical manifestation of the disease.

Indeed, recent observations suggest a role of SMN in tissues outside of the motor neuron with possible contribution to the pathology of SMA [9–14]. Notably, in a study designed to develop an SMA plasma protein biomarker panel to monitor disease progression and therapeutic efficacy, 84 putative markers regressing to motor function were found in patients (*n* = 108 total; type I = 17; type II = 49; type III = 42)) enrolled in the BforSMA study, including several markers of congestive heart failure and cardiovascular dysfunction [15]. Indeed, marked elevation of heart failure markers such as brain natriuretic peptide (BNP), fatty acid-binding protein 3 (FABP3), and creatine kinase (CK) in SMA patients suggests that normal cardiac function may require sufficient functional expression of SMN. Importantly, in vivo assessment of cardiovascular function indicates that SMA mice have impaired cardiac function [16]. Sympathetic denervation of the heart in SMA model mice [16–20] has been suggested as a cause of cardiac pathophysiology, but the mechanisms fully responsible for these cardiac deficits are unknown. Importantly, there have been no direct measurements of cardiomyocyte function in any SMA model so it has been difficult to conclude with certainty whether SMN directly impairs cardiac function.

We sought to determine if SMN deficiency compromises contractile function in ventricular cardiomyocytes isolated from the SMNΔ7 mouse (*SMNΔ7*;*SMN2*; *Smn*^−/−^) that recapitulates a severe form of SMA with an average life span of 14 days and muscle weakness onset at post-natal day 5 (P5) [21]. In this model, we observe significant elevation of heart failure marker expression as early as P5 and marked impairment of contractile function in isolated cardiomyocytes. Moreover, we observe marked reduction in SERCA2 expression and consequent slowed Ca^2+^ transient kinetics in cardiomyocytes isolated from SMA mice and generated from SMA patient-derived iPSCs. These results show that SMN deficiency also causes cardiac deficits directly in cardiac myocytes, potentially by altering SERCA2 expression and calcium cycling kinetics, in addition to any autonomic dysfunction.

## Materials and methods

### Generation of transgenic mice

The generation and phenotypic characterization of the SMN-deficient transgenic SMNΔ7 mouse line (*SMNΔ7*; *SMN2*; *Smn*^−/−^) has been described previously [22]. Briefly, the SMNΔ7 mouse line is a triple mutant model with a double copy of SMA cDNA lacking exon 7 and two copies of the human *SMN2* on an *Smn*^−/−^ background. SMNΔ7 mouse transgenic and unaffected littermate mice (post-natal day 5 (P5) and 10 (P10), both male and female) were used in the present study. All procedures complied with the standards for the care and use of animal subjects as stated in the *Guide or the Care and Use of Laboratory Animals* (NIH publication no. 85-23, revised 1996), and all protocols were approved by the IACUC at the Uniformed Services University of the Health Sciences.

### RNA extraction and quantification

Tissue (50–100 mg) was homogenized in 1 ml TRIzol reagent (Thermo Fisher, cat 15596018), and total RNA was isolated and converted to cDNA as previously described [23]. Gene-specific Taqman primer and probe sets were purchased from Applied Biosystems: atrial natriuretic peptide (ANP; *Nppa, Mm01255748_g1*), brain natriuretic peptide (BNP, *Nppb, Mm01255770_g1*), skeletal muscle actin (*Acta1, Mm00808218_g1*), SERCA2 (*Atp2a2, Mm01201431_m1*), hypoxanthine guanine phosphoribosyl transferase (*Hprt, Mm01318743_m1*), and glyceraldehyde 3-phosphate dehydrogenase (*Gapdh, Mm01180221_g1*). qRT-PCR reactions were performed in triplicate using the StepOnePlus™ Real-Time PCR System (Applied Biosystems). The following primers were purchased from Integrated DNA Technologies: *SERCA2*, 5′-TGAGACGCTCAAGTTTGTGG-3′; *SERCA2a*, 5′-ATGCAGAGGGCTGGTAGATG-3′; *SERCA2b*, 5′-ACAAACGGCCAGGAAATG-3′; and *GAPDH*, 5′-GCATGGCCTTCCGTGTTC-3′, 5′-ATGTCATCATACTTGGCAGGTTTC-3′. The level of each transcript was quantified by the threshold cycle (Ct) method using *Gapdh* and *Hprt* as endogenous controls.

Values were normalized to the mean of the unaffected group for each gene, which was assigned as 1.

### Transcriptome profiling by RNA sequencing

Total RNA was quantified via a fluorescence dye-based methodology (RiboGreen, Thermo Fisher, cat. R11490) on a Spectramax Gemini XPS plate reader (Molecular Devices, Mountain View, CA). RNA integrity was assessed using automated capillary electrophoresis on a Fragment Analyser (Advanced Analytical Technologies, Inc, Santa Clara, CA). Total RNA input of 200 ng was used for library preparation using the TruSeq Stranded mRNA Library Preparation Kit (Illumina, San Diego, CA, cat 20020594). Sequencing libraries were quantified by PCR using KAPA Library Quantification Kit for NGS (Roche, Wilmington, MA, cat KK4854) and assessed for size distribution on a Fragment Analyser. Sequencing libraries were pooled and sequenced on a NextSeq. 500 Desktop Sequencer (Illumina) using a NextSeq. 500 High Output Kit v2 with paired-end reads at 75 bp length. Raw sequencing data was demuxed using bcl2fastq2 Conversion Software 2.17 before alignment using TopHat Alignment v1.0 and differential expression analysis using Cufflinks Assembly and DE v1.1.0 on BaseSpace Onsite (Illumina). Functional enrichment analysis was performed using the PANTHER Classification System. Significantly overrepresented gene ontology biological processes at *p* < 0.01 were adjusted using a Bonferroni correction for multiple testing.

### Western blots

Fresh ventricular tissue was homogenized (Polytron) in RIPA lysis buffer (Sigma, cat R0278). Isolated proteins were separated by SDS-PAGE (4–15%, BioRad, cat 456-1084) and transferred onto PVDF membranes (BioRad, cat BR20191004). Blots were probed with the following specific antibodies: anti-SERCA2a antibody (1:1000, Cell Signaling Technology, cat 9580), anti-SMN (1:1000, BD Transduction Laboratories, cat 610647), anti-α-actin (1:5000 mybiosource.com, cat MBS477269) anti-BNP (1:1000, Abcam, cat ab236101), and GADPH (1:5000, Abcam, cat ab8245). Bound antibodies were detected with SuperSignal West Dura ECL substrate (Pierce, cat 34075).

### Cell contractility and calcium transient measurements in mouse cardiomyocytes

Unloaded cell shortening and intracellular calcium (Ca^2+^) transients were measured in freshly isolated ventricular myocytes, prepared as described previously [24, 25]. Isolated myocytes were transferred into a recording chamber mounted on an Olympus X51 inverted microscope and superfused with normal Tyrode solution (composition in mM: NaCl, 137; KCl, 5.4; NaH_2_PO_4_, 0.16; glucose, 10; CaCl_2_, 1.8; MgCl_2,_ 0.5; HEPES, 5.0; NaHCO_3_, 3.0; pH 7.3–7.4.). All experiments were performed at room temperature. Video images were acquired using a Myocam camera (IonOptix). Cells were field stimulated at 1 Hz in all experiments.

In experiments aimed at measuring intracellular Ca^2+^, isolated cells were incubated in Wittenberg Isolation Medium (WIM, composition in mM: NaCl, 116; KCl, 5.3; NaH_2_PO_4_, 1.2; glucose, 11.6; MgCl_2_, 3.7; HEPES, 20; l-glutamine, 2.0; NaHCO_3_, 4.4; KH_2_PO_4_, 1.5; 1X essential vitamins (GIBCO cat 12473-013);1X amino acids (GIBCO cat 11120-052); pH 7.3-7.4.) solution containing fluo-3-AM (1 μM) for 30 min at room temperature. Fluo-loaded cells were transferred into the recording chamber and perfused with normal Tyrode solution supplemented with 500 μM probenecid to inhibit dye export. Cells were stimulated to contract at 1 Hz in all cases and fluorescence was captured with a photomultiplier tube (IonOptix). Intracellular Ca^2+^ concentration ([Ca^2+^]_i_) was estimated using the Maravall equation [26]:

$$ \left[ Ca\right]= Kd\bullet \frac{\left(\raisebox{1ex}{$F$}\!\left/ \!\raisebox{-1ex}{$F\max $}\right.-\raisebox{1ex}{$1$}\!\left/ \!\raisebox{-1ex}{$ Rf$}\right.\right)}{1-\raisebox{1ex}{$F$}\!\left/ \!\raisebox{-1ex}{$F\max $}\right.} $$

Where *K*_d_ was assumed to be 600 nM [27] and *R*_f_ = *F*_max_/*F*_min_. *F*_min_ and *F*_max_ were measured in each cell by incubating the cell in modified Tyrode solutions supplemented with 20 mM 2,3 butadione monoxime, 10 mM A23187 and either 10 mM EGTA, or 100 mM CaCl_2_, respectively.

### Cell lines

To study the effects of SMN-deficiency on human cardiomyocytes, we utilized iPSCs from a spinal muscular atrophy patient (GM23240; Coriell). These iPSCs were reprogrammed from fibroblasts using lentiviral constructs encoding OCT4, SOX2, NANOG, and LIN28. NCRM-1 iPSCs were used as a control line and obtained from XCell Sciences (Novato CA). NCRM-1 iPSCs were obtained from XCell Sciences and reprogrammed by episomal plasmid from CD 34+ human cord blood cells. To grow and maintain iPSC lines, standard 6-well tissue culture plates were coated with growth factor reduced Matrigel (Corning; cat 354277 ) diluted 1:100 in DMEM/F12 (Gibco, cat 11330-032) same day as iPSC plating. Frozen stocks of iPSCs were thawed and plated on Matrigel coated plates in TeSR E8 (StemCell, cat 05990) supplemented with ROCK inhibitor (Y-27632, 10 μ M, Tocris, cat 1254). After 24 h, media was replaced with TeSR E8 (without ROCK inhibitor) and culture media was replaced daily until fully confluent. iPSC lines were passaged using 0.5 mM EDTA (Life Technologies, cat 15575-038) in PBS without CaCl_2_ and MgCl_2_ (Hyclone, Slt30256.01). Cells were maintained at 37 °C, 5% CO2.

### Generation and maintenance of cardiomyocytes

We began the cardiomyocyte differentiation protocol when cells were 80% confluent using the protocol developed by Feaster et al [28]. Briefly, differentiation was initiated (day 0–2) by replacing TeSR E8 medium with RPMI 1640 medium (Lonza, cat 12-702F) supplemented with B27 (minus insulin, Gibco, cat A1895601) and CHIR99021 (6 μM, LC Laboratories, cat C-6556), a GSK3 inhibitor. On days 3–4, CHIR99021 was removed and replaced with RPMI 1640 medium supplemented with B27 (minus insulin) and IWR-1 (500 μM, Sigma, cat I0761), a Wnt signaling inhibitor. On days 5–9, cells were maintained in RPMI 1640 medium supplemented only with B27 (minus insulin). From days 10–15, a metabolic selection protocol was employed using RPMI 1640 without glucose (Life Technologies, cat 11879) plus B27 without insulin. Following metabolic selection, cells were maintained in RPMI 1640 supplemented with B27 (Gibco, cat 17504-044) and 1% pen strep (Gibco, cat 10378-016). Beating cardiomyocytes were fed daily until day 20 when functional assays were carried out as described below.

### Transfection

To increase SMN expression in patient iPSC-derived cardiomyocytes, we transfected cells with SMN-GFP cDNA plasmid (1 μg) using lipofectamine 3000 (Invitrogen, cat 100022052) and P3000 (Invitrogen, cat 100022058) for 48 h according to the manufacturer protocols. Transfection was performed at day 18 of differentiation. Genartion of SMN-GFP plasmid was previously described [29] and a gift from Greg Matera (University of Carolina at Chapel Hill). To reduce SMN expression in control iPSC-derived cardiomyocytes, cells were transfected with 40 pmol of pre-validated siRNA (IDT, cat 37206943) and RNAi Max (Life Technologies, cat 56532) per manufacturer protocol at the 18 day time point. Opti-MEM (Gibco, cat 11058-021) diluent was used in both protocols. Successful transfection was confirmed in western blot analysis.

### Flow cytometry

SMA and Control patient iPSC-derived cardiomyocytes were dissociated using TrypLE (Gibco, cat 12605-010). Disaggregated cells were centrifuged and resuspended in fixation buffer (Biolegend, cat 554655) for 15 min at room temp, washed 3 times in 1X permeabilization wash buffer (Biolegend, cat 421002, and resuspended in permeabilization wash buffer containing FITC conjugated anti- Cardiac Troponin (1:20, Miltenyi Biotec, cat 130-106-689) for 1 h at room temp protected from light. Cells were read in cell staining buffer (Biolegend, cat 420201) on BD Accuri C6 Flow Cytometer CFlow Plus software. Unstained cells were used as a negative control.

### Calcium imaging

SMA and Control patient iPSC-derived cardiomyocytes wells were washed with PBS and incubated in normal Tyrode’s solution (composition in mM: NaCl, 137 mM; KCl, 5.4 mM; NaH_2_PO_4_, 0.16 mM; glucose, 10 mM; CaCl_2_, 1.8 mM; MgCl_2,_ 0.5 mM; HEPES, 10.0 mM; NaHCO_3_, 3.0 mM; pH 7.3–7.4.) supplemented with cell permeant Fluo-4 AM, fluorescent Calcium indicator (1 μM, Invitrogen, cat F14201) and probenecid (500 μM, Sigma, cat P8761). Cells were incubated for 15 min at 37 °C, 5% CO2. Calcium transients were recorded on a Nikon eclipse Ti2 inverted microscope equipped with a large view CMOS camera on a × 20 objective with NIS Elements AR software (Nikon). Decay at *T*_10_, *T*_50_, *T*_75_, *T*_90_, and *T*_100_ were analyzed using Clampfit software (Axon).

### Reagents

A complete list of reagents, primer sequences with source information can be found in Supplemental Table 2.

### Data analysis

All data were analyzed using IonWizard, Clampfit, and Microsoft Excel software and, except where noted, results are presented as means ± SEM (standard error of the mean). In all cases, *p* < 0.05 was considered significant. Statistical tests used and resultant *p* values are given in the Figure legends. Statistical analysis was performed in GraphPad Prism 7.

## Results

### Heart failure markers are elevated in SMN-deficient mice

**Fig. 1:**
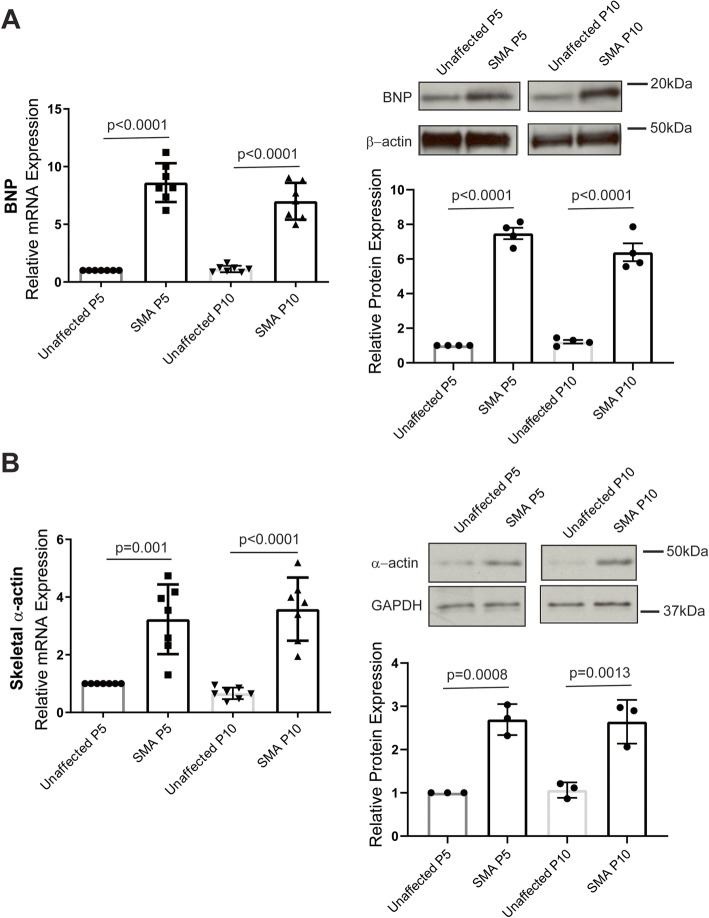
Elevated expression of heart failure markers in SMA model mice. qRT-PCR analysis of **a** BNP and **b** ACTA1 mRNA expression. Expression levels (mean ± SEM) in SMN-deficient (SMA) heart tissue are represented relative to unaffected controls at each time point. Statistical analysis: *p* values shown for one-way ANOVA, Tukey’s post hoc test, *n* = 7

There is growing evidence suggesting that peripheral tissues, including the heart, may be affected by the loss of SMN function in SMA patients. Heart failure is associated with the reactivation of fetal genes, including atrial natriuretic peptide (*ANP*; *Nppa*), brain natriuretic peptide (*BNP*; *Nppb*), and skeletal α-actin (*Acta1*), associated with structural and functional remodeling of the heart [30]. The activation of ANP and BNP in particular has been shown to correlate well with the clinical severity and prognosis of heart failure [31–33]. ANP and BNP mRNA expression was markedly increased in whole heart tissue from SMN deficient mice modeling a severe form of the disease [22] at both post-natal day 5 (P5; early in the disease progression) and post-natal day 10 (P10; closer to end-stage) when compared to unaffected littermates (Fig. 1: Supplemental Figure 1). Similarly, we found increased expression of skeletal α-actin at P5. Together, these results imply that mechanical function of the heart may be altered early in the disease progression of this severe SMA mouse model.

### Diastolic function is impaired in SMN-deficient mouse cardiomyocytes

**Fig. 2.**
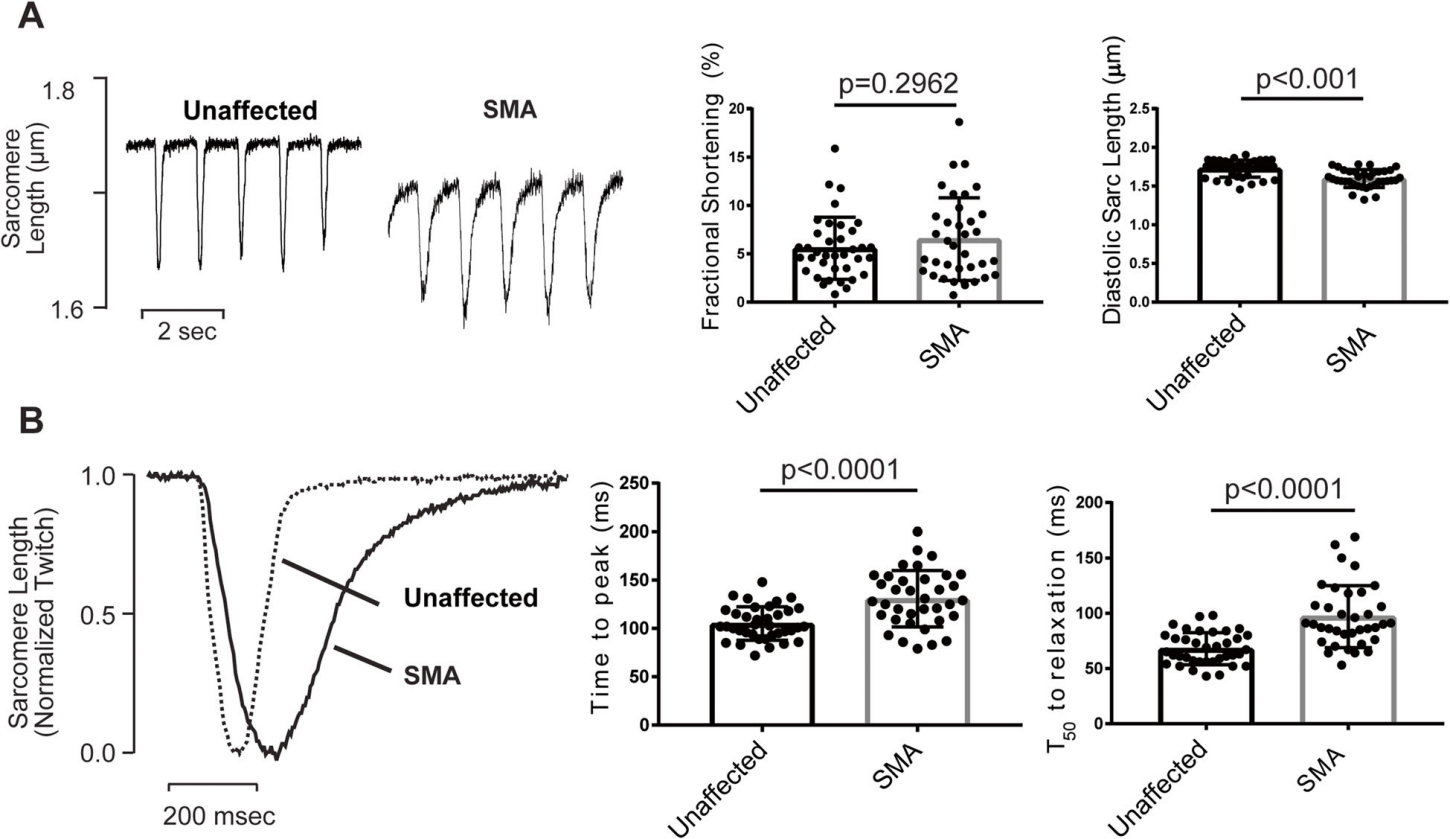
Contraction kinetics are slowed in SMA cardiomyocytes. **a** Representative recordings of sarcomere length obtained from ventricular myocytes isolated from unaffected or SMA mice (aged P12–13). Cells were field stimulated to contract at 1 Hz. Fractional shortening was not markedly different; however, there was a significant decrease in the diastolic sarcomere length. **b** Shown are normalized average record of a typical single twitch illustrating the slowed contraction and relaxation kinetics in SMA compared to control cardiomyocytes. Both the time to peak shortening and time to 50% relaxation (*T*_50_) were significantly increased in SMA myocytes compared to control Statistical analysis: *p* values shown for *t* test, *n* = 36 and 37 for SMA and control, respectively

To directly test cardiac function, we compared unloaded sarcomere shortening in paced (1 Hz) ventricular cardiomyocytes isolated from unaffected and SMN-deficient mice at P12–P13 (Fig. 2). The mean amplitude of sarcomere shortening and fractional shortening were similar in myocytes from control and SMN deficient littermates. While the extent of sarcomere shortening was unaffected by SMN deficiency, the rate of contraction and relaxation were both markedly slowed in SMN deficient cells. Both time-to-peak shortening (131 ± 5 vs.105 ± 3 ms, *p < 0.01, *t* test) and time-to-50% re-lengthening (97 ± 5 vs. 68 ± 2 ms, *p < 0.01, *t* test) were markedly increased in SMN-deficient cells compared with cells from unaffected littermates. We also noted a significant reduction of resting (diastolic) sarcomere length in SMA myocytes (1.60 ± 0.02 μm vs. 1.73 ± 0.02 μm in control, *n* = 36 and 37, respectively,*p < 0.01, *t* test). Thus, as predicted by the elevated heart failure markers, cardiomyocyte function, particularly during diastole, is compromised in SMN-deficient mice.

### Ca^2+^ handling gene expression is altered in SMN-deficient mouse heart

**Fig. 3:**
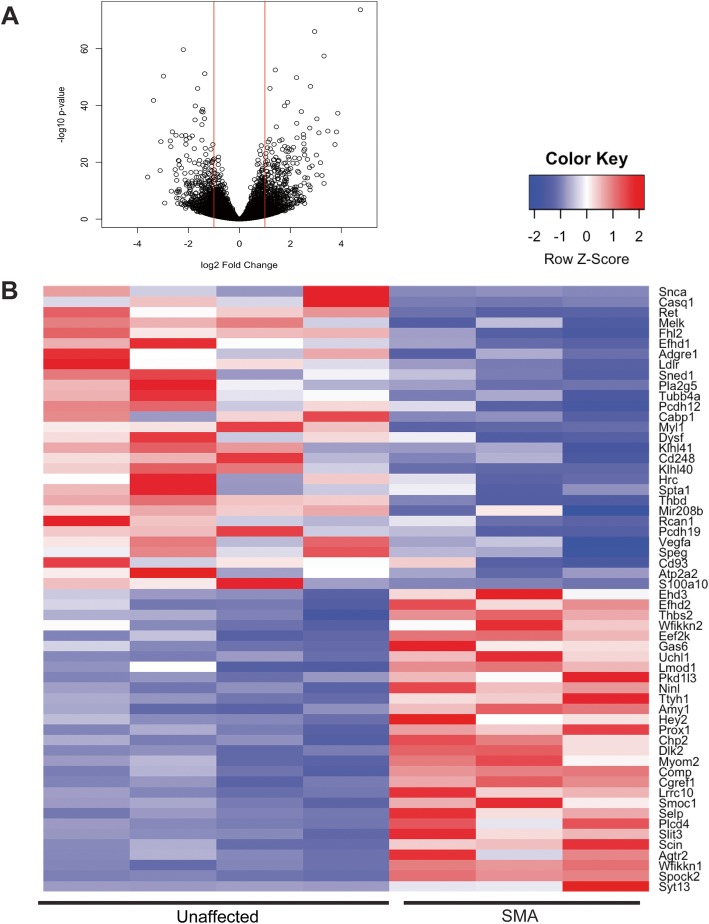
Transcriptome analysis suggests Ca^2+^ handling is altered in SMA heart. **a** Shown is a “volcano plot” of statistical significance versus transcript fold change in SMA compared to unaffected control hearts. A total of 1178 transcripts were up- or down-regulated in SMA heart. **b** Heat map illustrating a subset of genes that were significantly changed. Red denotes high expression, blue denotes low expression. Of note, the principal gene regulating Ca^2+^uptake into the sarcoplasmic reticulum, *Atp2a2* (SERCA2), was downregulated.

To gain an understanding of how SMN deficiency might affect cardiac function, we performed transcriptome expression profiling of whole heart tissue from SMA model mice at P10. Differential expression analysis identified 637 transcripts that were upregulated and 541 transcripts that were downregulated in heart tissue of SMA mice as compared to unaffected littermates (Fig. 3a; Supplemental Table 1). Subsequent analysis of the set of 1178 differentially expressed transcripts yielded significantly enriched gene ontologies for biological functions in regulation of muscle cell and fiber development (3.8-fold enrichment, *p* value = 3.9E−04). Additionally, specific key genes involved in muscle function and calcium-ion handling (1.8-fold enrichment, *p* value 1.6E−04) are lower (*Atp2a2*, *Casq1*, *CaBP1*, *S100a10*, *Rcan1*, and *Hrc*) and higher in transcript level (*Scin*, *Efhd2*, and *Myom2*) in heart tissue of SMA mice as compared to unaffected mice (Fig. 3b). Transcript expression of all identified calcium handling-related genes were significantly different prior to disease end-stage, suggesting that SMN-deficiency alters the cardiac transcriptome during disease progression.

### SERCA2 expression is reduced in SMN-deficient cardiomyocytes

**Fig. 4:**
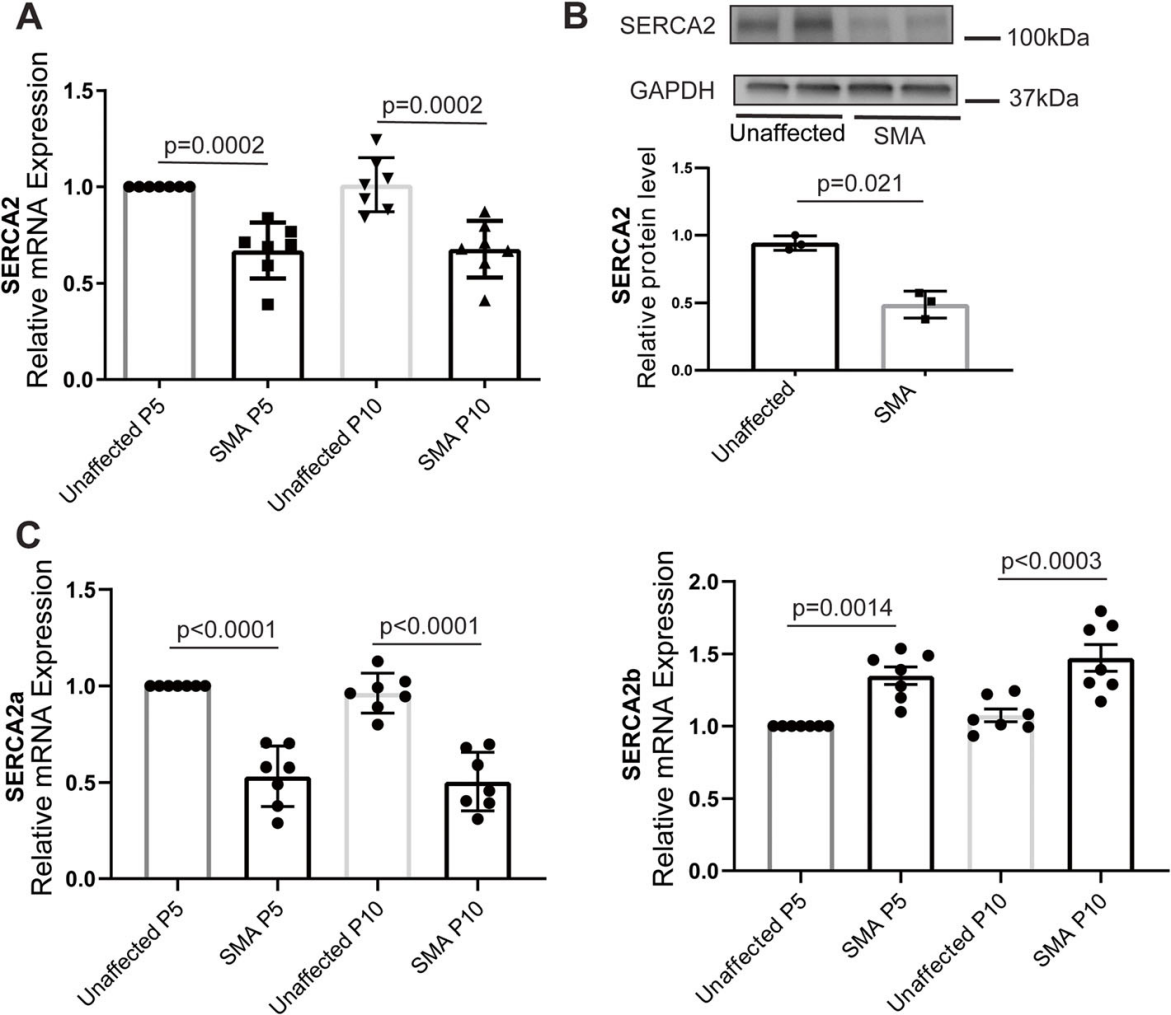
SERCA2a expression is reduced in SMA hearts. **a** qRT-PCR analysis of SERCA2 mRNA. Expression levels in SMN-deficient cardiomyocytes are represented relative to unaffected controls (*n* = 3). **b** Western blot analysis of SERCA2a protein levels. Quantification of SERCA protein level in SMA cells relative to controls at right. **c** qRT-PCR analysis of SERCA2a and SERCA2b mRNA. Expression levels in SMN-deficient (SMA) cardiomyocytes are represented relative to unaffected controls. Statistical analysis: p values shown for one-way ANOVA, Tukey’s post hoc test, *n* = 7

It is notable that *Atp2a2*, the gene encoding the sarcoplasmic reticulum (SR) Ca^2+^ ATPase or SERCA2, is found among the major Ca^2+^ handling related genes altered in the SMN-deficient heart. Impaired contractile function of the failing heart is often associated with dysregulation of intracellular Ca^2+^ cycling and reduced expression of SERCA2 protein [34–36]. As predicted from transcriptome data, we found that SERCA2 protein expression is reduced in SMN-deficient cardiomyocytes as early as P5 (Fig. 4). There are two major splice variants of SERCA2—SERCA2a and SERCA2b [37]. SERCA2a is considered the muscle specific isoform expressed in both cardiac muscle and slow twitch skeletal muscle, while the SERCA2b isoform is ubiquitously expressed. Given the role of SMN in regulating spliceosome assembly and function, we examined the relative expression of the two major splice variants in SMN-deficient and control hearts. Interestingly, there appears to be an isoform switch, with reduced SERCA2a and increased SERCA2b mRNA expression, but overall there is a significant reduction of SERCA2 protein regardless of the isoform.

### Ca^2+^ reuptake kinetics are slowed in SMN-deficient mouse cardiomyocytes

**Fig. 5:**
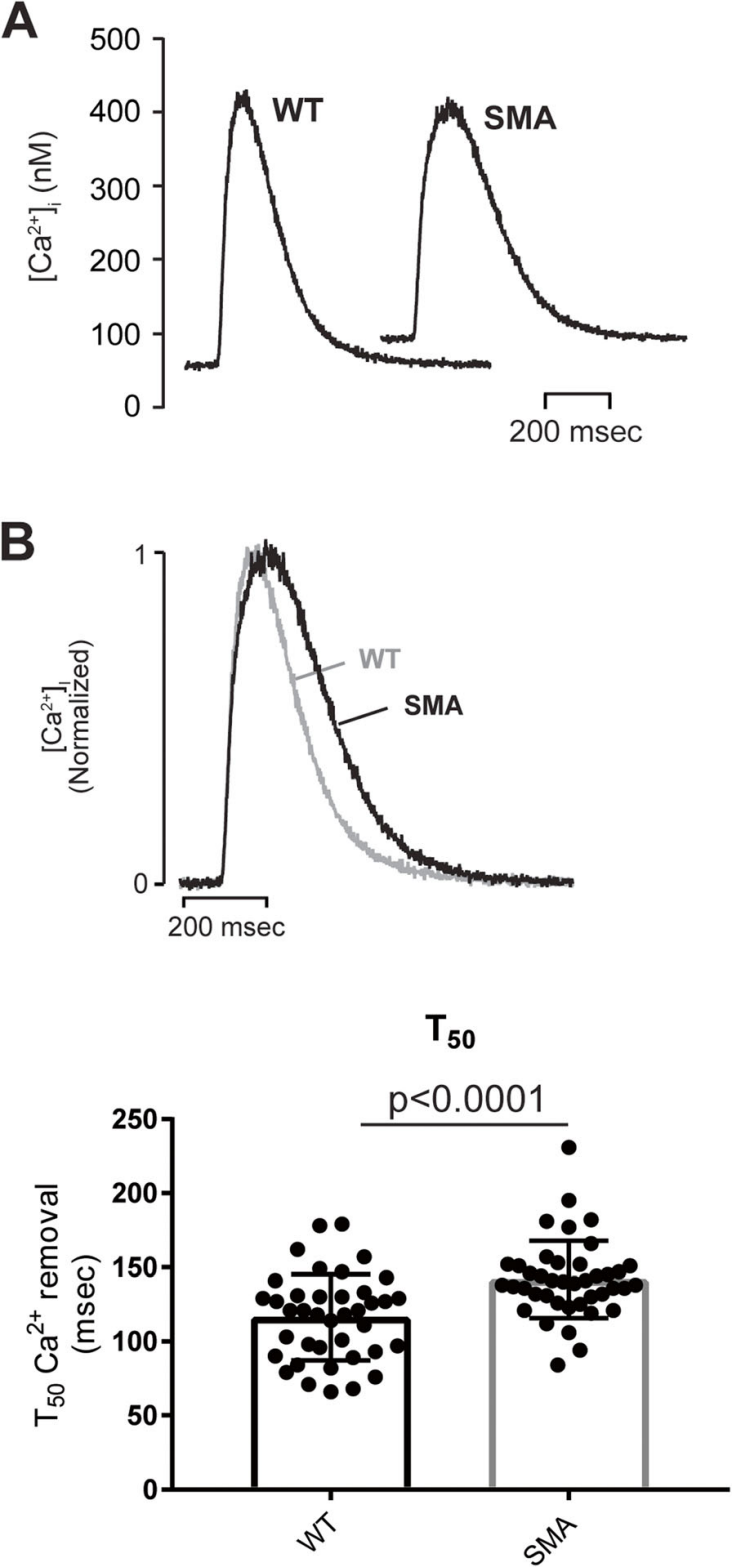
[Ca^2+^]_i_ transient kinetics are slowed in SMA cardiomyocytes. **a** Representative [Ca^2+^]_i_ transient measured in ventricular myocytes isolated from SMA or unaffected hearts (aged P12–13). There was no significant difference in the estimated diastolic or peak systolic [Ca^2+^]_i_ (*n* = 21 for both SMA and control). **b** Typical normalized [Ca^2+^]_i_ transients from SMA and control cells are shown illustrating that the time to 50% removal of cytoplasmic Ca^2+^ (*T*_50_) **c** was significantly increased in SMA myocytes compared to control in agreement with the slowed relaxation kinetics. Statistical analysis: *p* values shown for *t* test, *n* = 41 and 39 for SMA and control, respectively

SERCA2 is the major determinant of Ca^2+^ sequestration from the cytoplasm in murine cardiomyocytes [38–40]; therefore, it is predicted that SMN-deficient cells exhibit slowed removal of cytosolic Ca^2+^ following a triggered release. To test this, we estimated [Ca^2+^]_i_ during triggered Ca^2+^ transients in fluo-3-AM loaded ventricular myocytes isolated from unaffected and SMN-deficient littermates at P12-P13 (Fig. 5). Although resting [Ca^2+^]_i_ tended to be elevated prior to stimulation, there were no significant differences in diastolic or peak systolic [Ca^2+^]_i_ between control and SMA cardiomyocytes. Consistent with reduced expression of SERCA2, however, there was marked slowing of Ca^2+^ removal from the cytoplasm with *T*_50_ significantly (*p < 0.01, *t* test) increased in SMN cardiomyocytes (146 ± 4 ms) compared with control cells (126 ± 4 ms). These data are consistent with the conclusion that SMN-deficiency is associated with reduced SERCA2 expression, causing slowed Ca^2+^ reuptake and impaired cardiomyocyte mechanical function.

### SMN deficiency slows [Ca^2+^]_i_ reuptake kinetics and reduces SERCA2a expression in human iPSC-derived cardiomyocytes

**Fig. 6:**
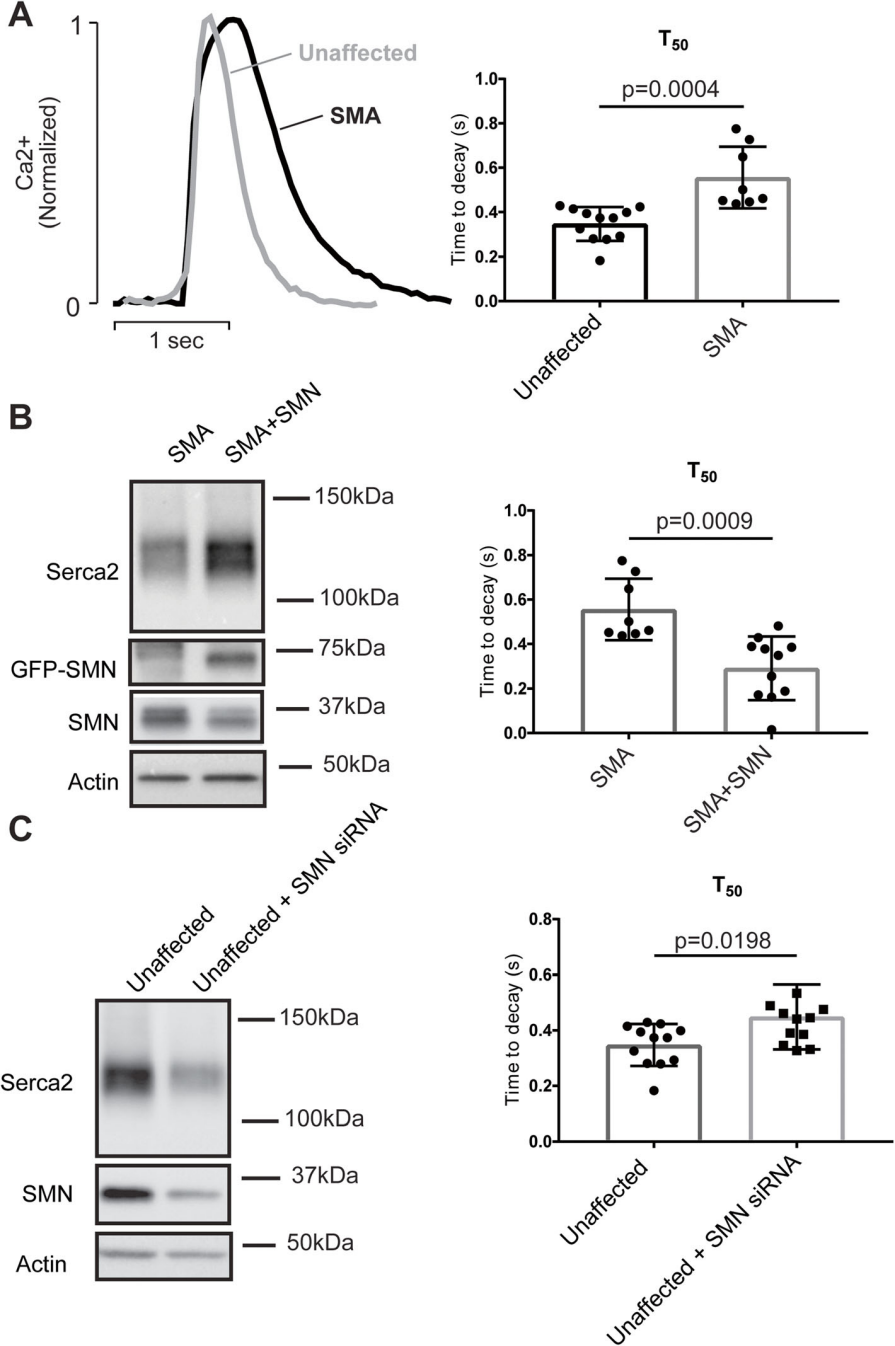
Defects in [Ca^2+^]_i_ handling are recapitulated in cardiomyocytes derived from SMA patient iPSCs. **a** Representative [Ca^2+^]_i_ transients measured in iPSC-derived cardiomyocytes engineered from unaffected control (NCRM1) and SMA patient (GM23240) iPSCs illustrating the slowed removal of cytoplasmic Ca^2+^. **b** Expression of SERCA2a expression is increased (western blot, *left*) and *T*_50_ is decreased (*right*) in SMA patient iPSC-derived cardiomyocytes when SMN protein is restored. Cells were transfected on day 18 of differentiation and recordings were made on day 20. **c** Similarly, expression of SERCA2a expression is reduced (western blot, *left*) and *T*_50_ is increased (*right*) in unaffected cotnrol iPSC-derived cardiomyocytes when SMN protein is reduced. Cells were transfected on day 18 of differentiation and recordings were made on day 20. Statistical analysis: *p* values shown for one-way ANOVA, Tukey’s post hoc test, *n* = 7

Human-induced pluripotent stem cells (iPSCs) have emerged as a powerful system to model human cardiac disease and study Ca^2+^ handling [41–44]. To confirm that our findings are not restricted to the mouse model, we assessed Ca^2+^ transients in spontaneously contracting clusters of cardiomyocytes generated from patient-derived (SMA) and unaffected control iPSCs. As expected, SMN protein expression was markedly reduced in SMA patient-derived cells. In agreement with data obtained from mouse cardiomyocytes, there was also marked slowing of Ca^2+^ reuptake kinetics in SMA cells with *T*_50_ (556 ± 49 ms) increased compared with control (348 ± 21 ms) (Fig. 6a). Moreover, transfection of patient-derived cells with SMN to increase SMN levels, increased both SERCA2 protein expression and Ca^2+^ reuptake rate (Fig. 6b). Conversely, using siRNA to reduce SMN expression in control cells, reduced both SERCA2 expression and the *T*_50_ for Ca^2+^ reuptake (Fig. 6c; Supplemental Figure 2). However, we did not observe the isoform switch from SERCA2a to SERCA2b, as observed in the mouse tissue, in the SMN-deficient IPSC-derived cardiomyocytes. Taken together, these results demonstrate that SMN regulates SERCA2 expression and intracellular Ca^2+^ cycling kinetics in cardiomyocytes that may impair cardiac function and lead to elevation of heart failure markers, as observed in mice (Fig. 1) and patients with SMA [15].

## Discussion

The major pathological feature of SMA is neuromuscular degeneration; however, there have been reports that cardiovascular function is impaired in both SMA patients and mouse models of the disease [16, 17, 45–47]. The data in the present study demonstrate increased expression of heart failure molecular markers, reduced expression of SERCA2, and impairment of cardiomyocyte contraction in a severe mouse model of SMA. Moreover, these findings are reversibly recapitulated in cardiomyocytes generated from patient-derived iPSCs, indicating that SMN deficiency likely causes a similar phenotype in human cardiac muscle as well. Taken together, these results add support to the conclusion that cardiovascular function is compromised by loss of SMN function and indicate that dysregulation of SERCA2 expression and Ca^2+^ homeostasis lies at the center of cardiac pathophysiology.

### Heart function and failure in SMA

Efforts to treat SMA are largely focused on preventing or reversing neuromuscular degeneration. We showed previously that daily injections of the HDAC inhibitor, trichostatin A, improves motor function and modestly extend the lifespan of SMA mice [48]. Interestingly, however, these mice ultimately die from cardiovascular causes [48]. Here, we show that BNP and skeletal α-actin are elevated as early as P5 in SMA mice, prior to the development of profound neuromuscular degeneration. Moreover, even though cell shortening and Ca^2+^ transient amplitude are preserved in SMN-deficient cells, we show that the rate of relaxation and diastolic sarcomere length are markedly reduced in SMA cardiomyocytes, suggesting that SMN deficiency may be associated with diastolic heart failure or heart failure with preserved ejection fraction [49]. It is interesting to note, that SMA patients often require artificial ventilation as a result of lung congestion and respiratory distress even though the diaphragm muscle is relatively spared from degeneration [50]. Although speculative, heart failure resulting from SMN deficiency as evidenced here may contribute to the respiratory pathology in SMA patients in addition to neuromuscular degeneration. Nevertheless, the present findings support the conclusion that cardiac function is compromised in SMN deficient animals, even though the major pathological feature is neuromuscular degeneration.

### Effects of SMN deficiency appear to be cell autonomous

Because neurodegeneration is central to SMA pathology, it is reasonable to consider the influence of innervation of the heart as a cause of pathology. Indeed, several studies suggest that sympathetic denervation and sympatho-vagal imbalance are evident in SMA patients and mouse models of the disease [16–18, 51]. Bradycardia and slowed conduction has been reported in patients, consistent with sympathetic denervation of the heart [19, 20]. We cannot rule out that sympathetic denervation contributes to cardiovascular pathology in SMA; however, here we show clear evidence of impaired cardiac function with slowed intracellular Ca^2+^ dynamics and reduced SERCA2 expression in a model of severe, type I SMA. It is likely that the degree of cardiac involvement varies with disease severity and a study in patients with the less severe type II/III SMA variant reported no evidence of cardiac dysfunction [45]. Importantly, the reduction in SERCA2 expression and slowing of Ca^2+^ reuptake was recapitulated and reversible in cardiomyocytes generated from SMA patient-derived iPSCs. When coupled with the observation that evidence of heart failure emerges as early as P5 in the mouse, this result provides strong evidence that the impairment of cardiomyocyte function is a result of cell autonomous loss of SMN function, and not secondary to neurodegeneration.

### Altered Ca^2+^ handling as a common disease mechanism in SMA

It remains unclear why SMN deficiency so potently targets motor neurons for degeneration. The principal finding in this study is that SMN deficiency causes a decrease in the expression of SERCA2 protein in both SMA mouse heart and cardiomyocytes generated from patient-derived iPSCs. SMN is known to play a role in snRNP biogenesis and spliceosome assembly [6–8]. It is not known how SMN deficiency inhibits SERCA2 biosynthesis, but we did observe an apparent isoform switch, with reduced SERCA2a and increased SERCA2b mRNA expression albeit with a significant reduction in total SERCA2 protein regardless of the isoform. One possibility is that disruption of alternative splicing mechanisms in cardiomyocytes leads to reduced SERCA2 expression. However, the SERCA2 alternative splicing was not observed in the human cardiomyocytes when SMN was knocked down even though total SERCA2 expression was reduced. This suggests that alternative splicing cannot fully account for the reduced SERCA2 expression in SMN-deficient cells.

Interestingly, SMN deficiency also affects intracellular Ca^2+^ handling in motor nerve terminals. Similar to the observations in the present study, Ca^2+^ removal from the cytoplasm is significantly impaired in motor neuron terminals following repeated stimulation [52]. Elevated basal Ca^2+^ has also been observed in SMN-deficient iPSC-derived astrocytes [53], and there is also a report showing reduced SERCA1a protein in hindlimb muscles of SMN-deficient mice [54]. These findings coupled with the data in the present study suggest that dysregulation of Ca^2+^ may be a common disease mechanism in SMA.

## Conclusion

Collectively, these results show for the first time that SMN deficiency alters intracellular Ca^2+^ signaling and cardiac excitation-contraction coupling in a model of SMA. These findings are recapitulated in SMA patient IPSC-derived cardiomyocytes suggesting that this is a cell autonomous outcome of SMN deficiency and suggest that dysregulation of intracellular Ca^2+^ may be a common pathological mechanism in the disease. Finally, while neuromuscular degeneration remains the hallmark feature of the disease, impaired heart function may be a contributing factor in disease progression that will require monitoring in light of new therapies that are improving motor function and extending survival.

## Supplementary information

**Additional file 1:Supplemental Figure 1.** Elevated expression of ANP in SMA model mice. qRT-PCR analysis of ANP mRNA expression. Expression levels (mean ± SEM) in SMN-deficient (SMA) heart tissue are represented relative to unaffected controls at each time point. Statistical analysis: p values shown for one-way ANOVA, Tukey’s post hoc. **Supplemental Figure 2.** Serca2 levels reduced in cardiomyocytes derived from SMN-deficient iPSCs. Expression of SERCA2a expression was determined in control and SMN-deficient iPSC-derived cardiomyocytes. Cells were transfected with siRNA targeting SMN on day 18 of differentiation and qRT-PCR performed 48 hours after transfection. Values represent mean ± SEM.

**Additional file 2.** Table 1 Transcriptome analysis.

**Additional file 3.** Table 2 Reagents

## Data Availability

All data generated or analyzed during this study are included in this published article.

## Abbreviations

SMA: Spinal muscular atrophy
SMN: Survival motor neuron
SERCA: Sarco/endoplasmic reticulum Ca^2+^-ATPase
iPSC: Induced pluripotent stem cell
BNP;Nppp: Brain natriuretic peptide
Acta1: Skeletal α-actin
P: Post-natal
