# Supplementary material for: SMN-deficiency disrupts SERCA2 expression and intracellular Ca2+ signaling in cardiomyocytes from SMA mice and patient-derived iPSCs
Source: Skelet Muscle. 2020 May 8;10:16. doi: 10.1186/s13395-020-00232-7 (PMC7206821; doi:10.1186/s13395-020-00232-7)
Supplement: Supplementary file 1 — Additional file 1:Supplemental Figure 1. Elevated expression of ANP in SMA model mice. qRT-PCR analysis of ANP mRNA expression. Expression levels (mean ± SEM) in SMN-deficient (SMA) heart tissue are represented relative to unaffected controls at each time point. Statistical analysis: p values shown for one-way ANOVA, Tukey’s post hoc. Supplemental Figure 2. Serca2 levels reduced in cardiomyocytes derived from SMN-deficient iPSCs. Expression of SERCA2a expression was determined in control and SMN-deficient iPSC-derived cardiomyocytes. Cells were transfected with siRNA targeting SMN on day 18 of differentiation and qRT-PCR performed 48 hours after transfection. Values represent mean ± SEM. [file 13395_2020_232_MOESM1_ESM.docx]

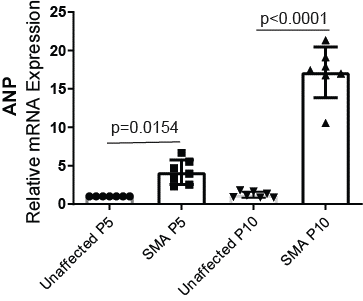


**Supplemental Figure 1.**  **Elevated expression of ANP in SMA model mice.** qRT-PCR analysis of ANP mRNA expression. Expression levels (mean ± SEM) in SMN-deficient (SMA) heart tissue are represented relative to unaffected controls at each time point. Statistical analysis: p values shown for one-way ANOVA, Tukey’s post-hoc test, n = 7.


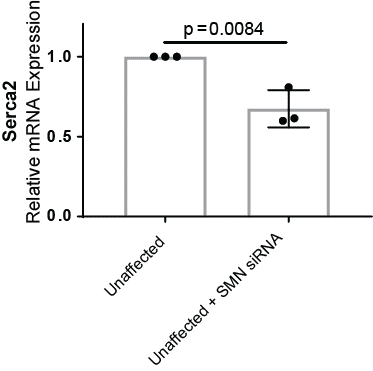


**Supplemental Figure 2:** Serca2 levels reduced in cardiomyocytes derived from SMN-deficient iPSCs. Expression of SERCA2a expression was determined in control and SMN-deficient iPSC-derived cardiomyocytes. Cells were transfected with siRNA targeting SMN on day 18 of differentiation and qRT-PCR performed 48 hours after transfection. Values represent mean ± SEM.
