# Supplementary material for: SMN-deficiency disrupts SERCA2 expression and intracellular Ca2+ signaling in cardiomyocytes from SMA mice and patient-derived iPSCs
Source: Skelet Muscle. 2020 May 8;10:16. doi: 10.1186/s13395-020-00232-7 (PMC7206821; doi:10.1186/s13395-020-00232-7)
Supplement: Supplementary file 3 — Additional file 3. Table 2 Reagents [file 13395_2020_232_MOESM3_ESM.docx]

| **Item Name** | **Company** | **Catalog Number** |
| --- | --- | --- |
| TRIzol reagent | Thermo Fisher | 15596018 |
| ANP; Nppa Taqman Probe | Applied Biosystems | Mm01255748_g1 |
| BNP, Nppb Taqman Probe | Applied Biosystems | Mm01255770_g1 |
| Acta1 Taqman Probe | Applied Biosystems | Mm00808218_g1 |
| Atp2a2 Taqman Probe | Applied Biosystems | Mm01201431_m1 |
| Hprt Taqman Probe | Applied Biosystems | Mm01318743_m1 |
| Gapdh Taqman Probe | Applied Biosystems | Mm01180221_g1 |
| RNeasy Mini Kit | Qiagen | 74106 |
| Quant-iT RiboGreen RNA Assay Kit | Thermo Fisher | R11490 |
| TruSeq Standed mRNA Library Preparation Kit | Illumina | 20020594 |
| KAPA Library Quantification Kit | Roche | KK4854 |
| RIPA lysis buffer | Sigma | R0278 |
| SDS-PAGE (4-15%) gel | BioRad | 456-1084 |
| PVDF membrane | BioRad | BR20191004 |
| anti-SERCA2 antibody | Cell Signaling Technology | 9580 |
| anti-SMN antibody | BD Transduction Laboratories | 610647 |
| anti-GAPDH antibody | Abcam | ab8245 |
| SuperSignal West Dura ECL substrate | Pierce | 34075 |
| fluo-3-AM | Invitrogen | F1242 |
| Probenecid | Sigma | P8761 |
| SMA patient iPSC cell line | Coriell | GM23240 |
| Control patient iPSC cell line | XCell Sciences | NCRM-1 |
| Matrigel | Corning | 354277 |
| DMEM/F12 | Gibco | 11330-032 |
| TeSR E8 | StemCell | 05990 |
| Y-27632 | Tocris | 1254 |
| 1x PBS without CaCl2 and MgCl2  0.5M EDTA | Hyclone  Life Technologies | Slt30256.01  15575-038 |
| B27 minus insulin | Gibco | A1895601 |
| CHIR99021 | LC Laboratories | C-6556 |
| IWR-1 | Sigma | I0161 |
| RPMI 1640 without glucose | Life Technologies | 11879 |
| RPMI 1640 | Lonza | 12-702F |
| B27 supplement | Gibco | A1895601 |
| pen strep | Gibco | 1037-016 |
| SMN-egfp | Addgene | 37057 |
| Lipfectamine 3000 | Invitrogen | 100022052 |
| P3000 | Invitrogen | 100022058 |
| SMN siRNA | IDT | 37206943 |
| RNAi Max | Life Technologies | 56532 |
| Opti-MEM | Gibco | 11058-021 |
| TrypLE | Gibco | 12605-010 |
| Fixation Buffer | Biolegend | 554655 |
| Permeabilization wash buffer | Biolegend | 421002 |
| FITC anti-Cardiac Troponin | Miltenyi Biotec | 130-106-689 |
| Fluo-4 AM Calcium indicatior | Invitrogen | F14201 |
| Cell Staining Buffer | Biolegend | 420201 |

SYBR Green Primer Sequences

| **mRNA Target** | **Species** | **Forward Primer Sequences** | **Reverse Primer Sequences** |
| --- | --- | --- | --- |
| *SERCA2a* | Human | 5′-TGAGACGCTCAAGTTTGTGG-3′ | 5′-ATGCAGAGGGCTGGTAGATG-3′ |
| *SERCA2b* | Human | 5′-TGAGACGCTCAAGTTTGTGG-3′ | 5′-ACAAACGGCCAGGAAATG-3′ |
| *GAPDH* | Human | 5′-GCATGGCCTTCCGTGTTC-3′ | 5′-ATGTCATCATACTTGGCAGGTTTC-3′ |
